# Health Effects of Methylmercury in Coastal Areas of the Yatsushiro Sea, Far from Minamata

**DOI:** 10.3390/toxics12100751

**Published:** 2024-10-16

**Authors:** Shigeru Takaoka, Tadashi Fujino, Shin-ichi Shigeoka, Yaeko Itai

**Affiliations:** 1 Kyoritsu Neurology and Rehabilitation Clinic, 2-2-28 Sakurai-cho, Minamata 867-0045, Japan; 2 Minamata Kyoritsu Hospital, 2-2-12 Sakurai-cho, Minamata 867-0045, Japanshigeoka@mk-kyouritu.com (S.-i.S.);

**Keywords:** methylmercury, prevalence of sensory disturbance, attributable fraction, quantitative sensory examination, cortical sensory disturbance

## Abstract

Minamata disease, caused by ingesting seafood contaminated with methylmercury dumped by corporations, was discovered in 1956; however, there has been no continued investigation to determine the full extent of the damage. Since 2004, it has been discovered that affected patients can be found in areas further away from Minamata than previously known. In the present study, we investigated various symptoms and somatosensory disturbances in western Miyanokawachi District, northern Himedo District, southwestern Nagashima District, and the uncontaminated Amami district and calculated the proportion of patients with sensory disturbances as a percentage of the population in each area. Both touch and pain sensations, with a predominance of the peripheral extremities, were observed in 58.6% of patients in Miyanokawachi, 53.9% in Himedo, 37.8% in Nagashima, and 1.4% in Amami. The lowest estimates of attributable fractions of the exposed group for four-limb-predominant sensory disturbance in the population of the contaminated districts were 94.1% in Miyanokawachi, 94.6% in Himedo, and 91.4% in Nagashima, and general and perioral sensory disturbances were also high. This suggests that the presence or absence of these sensory disturbances is useful in the diagnosis of Minamata disease, even in more distant parts of the Yatsushiro Sea area.

## 1. Introduction

Minamata disease occurred in large numbers of residents who consumed fish and shellfish contaminated with methylmercury compounds that were discharged into the Yatsushiro Sea by the Chisso Corporation. Mercury discharges continued from 1932 to 1968, and contamination of the Yatsushiro Sea persisted thereafter [1]. It has been found that the contamination of fish and shellfish was widespread throughout the Yatsushiro Sea [2] (“Shiranui Sea” is another name for “Yatsushiro Sea”, and the “Shiranui Sea” referred to in our previous paper [2] is the same sea area as the “Yatsushiro Sea”).

The existence of Minamata disease was officially confirmed in 1956, but even after that, the health status of coastal residents was not sufficiently investigated. Dr. Tokuomi of Kumamoto University advocated for a theory about the end of Minamata disease in 1960 without conducting sufficient epidemiological surveys [3]. As a result, Minamata disease has been neglected, and only a small proportion of those with severe symptoms of Hunter–Russell syndrome have been recognized as patients.

After an outbreak of Minamata disease was confirmed in 1965 in the Agano River basin in Niigata Prefecture—a region far from Minamata in Japan—epidemiological surveys were conducted in this area, which revealed the presence of milder cases of Minamata disease, including those showing only sensory disturbance [4]. Subsequent research has also shown that Minamata disease is not a necessary condition for the presence of all the symptoms of Hunter–Russell syndrome [5,6] if methylmercury toxicosis from seafood is defined as Minamata disease.

In 1971–72, a survey conducted by the Department of Neuropsychiatry at Kumamoto University, which identified Minamata City and the Goshonoura Islands off the Yatsushiro Sea in Minamata as contaminated areas and Ariake Town facing the Ariake Sea as a control area, also identified fewer patients in the Goshonoura Islands than in Minamata [7]. However, more patients were subsequently identified in the Goshonoura Islands than at this time.

The Kumamoto Prefectural Government survey was conducted in 1971–74, but the results were only made public in 2015, and only 158 patients were identified as a result [8]. The fact that more than 1790 people were subsequently recognized in Kumamoto Prefecture suggests that the capture rate was extremely low and that the survey itself was problematic, but no further investigation was ever conducted.

In the diagnosis of methylmercury toxicosis in Japan, epidemiological information, such as the attributable fraction of the exposed group, has not been used at all, and the diagnosis was arbitrarily made by some neurologists and administrators. The 1977 criteria only considered those with severe symptoms of Hunter–Russell syndrome as Minamata disease patients [9,10]. The Ministry of the Environment ignored its own criteria, stating that if there is a 50% or greater probability of Minamata disease (which can be statistically determined), then it is possible to be diagnosed with Minamata disease.

**Figure 1:**
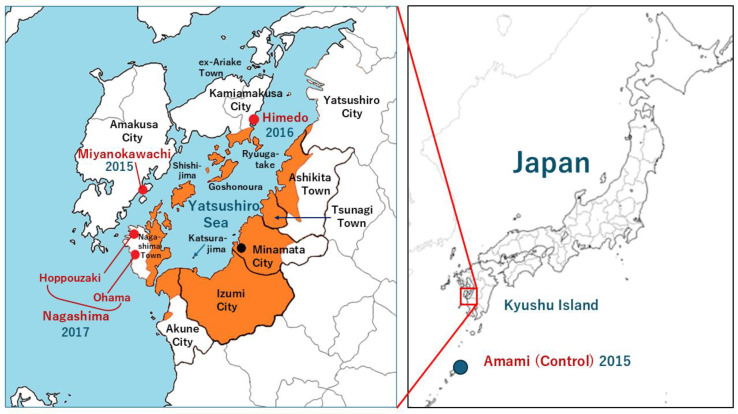
The surveyed Yatsushiro Sea (contaminated areas) and control areas.

In 2004, after the Japanese government’s policy of Minamata disease was rejected by the Supreme Court, a large number of residents began to undergo medical examinations, and the reality of the disease gradually became clear [2,11]. The contaminated area of the Yatsushiro Sea is the area shown in Figure 1. Until the 2000s, areas further away from Minamata City were not fully investigated, so the areas where Minamata disease was confirmed were as far west as the Goshonoura Islands [12], as far north as southern Yatsushiro City and Ryuugatake Town, and as far south as northeastern Nagashima and parts of Akune and Izumi Cities [5]. These are considered designated areas of contamination by the government and are shown in orange in Figure 1. Most of the attributable fraction of the exposed group for the sensory disturbance of the four limbs in these coastal populations studied so far was above 90% [5,6,12,13].

However, since 2005, patients have been recognized in the areas west of the Goshonoura Islands, north of the Ryuugatake District, and south of the southwestern part of Nagashima [2]. Although examination of those who wanted to be examined in these areas revealed the presence of residents with symptoms of Minamata disease, the proportion of patients with such health problems as a percentage of the population and the attributable fraction of the exposed groups had not been investigated. We conducted this study to investigate the prevalence of the various symptoms and sensory disturbances caused by methylmercury exposure in areas further away from Minamata.

## 2. Materials and Methods

### 2.1. Subjects, Covered Areas, and Population

Three study areas were selected as remote contaminated areas, which are further away from Minamata than the areas that have been recognized by the government as contaminated areas (designated areas: orange area in Figure 1).

The selected areas were Miyanokawachi District in Amakusa City, located west of the Goshonoura Islands as seen from Minamata; Himedo District in Kami-Amakusa City, north of the Ryuugatake District; and Hoppouzaki District, west of Nagashima Town, and Ohama District, south of Nagashima Town (collectively referred to as Nagashima District; see Figure 1). These three remote areas have not been recognized as contaminated by the government. Yamato Village on Amami Oshima Island, Kagoshima Prefecture, was selected as a control area.

Miyanokawachi District was surveyed on 31 October and 1 November 2015 for 206 residents who were born before December 1968 (electoral roll: end of March 2015). Some of the residents were surveyed between 30 October and early November.

In Himedo District, 198 residents who were born before December 1968 (electoral roll: end of March 2015) were surveyed on 9 and 23 October 2016.

In Nagashima District, a total of 128 residents of both Hoppouzaki (42) and Ohama (86) Districts (electoral roll: end of July 2017) who were born before December 1968 were surveyed on 4 November 2017 in Hoppouzaki District and on 5 November and 2 December 2017 in Ohama District.

In the Amami control area, 1043 residents of Yamato Village, Oshima County, who were born before December 1968 (statistics of residents of the town office, 31 October 2015) were surveyed on 22 and 23 November 2015.

In all four districts, subjects were recruited by stating that they would be examined for subjective symptoms and sensory abnormalities related to the effects of methylmercury.

### 2.2. Questionnaire Interview Using a Survey Form

The questionnaire included residential history (current address; whether or not they had lived in the same area between 1953 and 1968; whether or not they had lived in the designated areas); occupational history (in particular, whether or not they had worked in the fishing or seafood-handling industries); methods of obtaining seafood, seafood preferences, frequency of consumption, and daily intake; family occupational history and history of application for Minamata disease certification; and medical history.

For alcohol and smoking history, the subjects were asked about past and current consumption. For complications, participants were asked about the presence or absence of diabetes, stroke, vertebral or spinal cord disease, vibration disease, and other neurological disorders.

For subjective symptoms, subjects were asked to select “always”, “sometimes”, “used to”, or “never” for 58 symptoms related to sensory disturbances of the five senses, motor disturbances, body pain, general complaints, and psychological and intellectual problems. The criteria were determined by the way the person felt. Prior to the screening, subjects were asked to complete the questionnaire, but those who were unable to complete it independently were interviewed to complete it.

### 2.3. Sensory Function Tests

The neurological findings included standard touch and pain tests, thresholds of minimal tactile sense using a Semmes–Weinstein monofilament (see description in Section 2.3.3), and vibration thresholds using a C-tuning fork (see description in Section 2.3.4). The numbers of doctors who examined the patients were nine in Miyanokawachi, nine in Himedo, six in Nagashima, and eight in Amami: a total of three doctors participated in all four districts, three doctors participated in three districts, two doctors participated in two districts, and five doctors participated in only one district. All were experienced in examining patients with methylmercury poisoning and were fully informed of the test procedure before the examination.

During the sensory examination, the subject was allowed to be clothed, but the chest and peripheral areas beyond the shoulder and hip joint were left bare. The subject was asked to lie supine, relaxed, with eyes closed, and was instructed to “answer as you feel it”.

The timing of the subject’s responses during the sensory testing was emphasized. Consideration was given to withholding judgment when the responses were unnatural, when certain tests were exceptionally poor, when the responses were slow or ambiguous, when the responses were inconsistent, or when the subject was considered mentally unstable.

#### 2.3.1. Standard Touch Examination

A brush was used for the touch test. The brush was lightly brushed against the skin. Only the tip of the brush was lightly brushed against the chest to see if touch was clearly perceived. If touch disturbance was suspected, the brush tip was applied more lightly and touched to a level that would normally be considered tactile; if it could not be detected, the chest was considered to have touch disturbance.

The chest and the dorsal hand were then compared by stroking the chest and the dorsal hand with the brush. At this point, instead of asking, “Which is stronger?” they were asked, “Are they equal, or is one stronger than the other?”. If the response was slow or ambiguous, they were asked, “Are they almost equal?” or, after stimulating several points, they were asked, “How about on average?” Areas that were considered depressed were marked with shaded lines in the person-shaped diagram in the medical record.

If the dorsal hand (foot) was weaker than the chest, the chest was compared to the forearm (lower leg). If the forearm (lower leg) was weaker than the chest, the chest and upper arm (thigh) were compared. If the chest and dorsal hand (foot) were equal in degree or the dorsal hand (foot) was stronger, the chest was compared to the fingers of the hand for at least the index, middle, and ring fingers.

The chest and perioral (the bilateral upper and lower sides of the mouth) were compared. Comparisons were made between the perioral and buccal areas and between the perioral and forehead areas. When the touch disturbance was considered irregular, the intensity was checked in addition to the above procedure, and a shaded line was marked on the person-shaped diagram in the medical record.

#### 2.3.2. Standard Pain Examination

A locked pain needle was used for the pain test. The painful needle was applied to the chest without pressing it, using only the weight of the painful needle by gravity (20 g) and rated on four levels: 1. Painful, 2. Prickly, 3. Not prickly, and 4. No sense of touch. The pain point was checked by applying it to at least three places, as sometimes it would hit the pain point, and sometimes it would not.

The following judgments were made regarding the presence or absence of pain disturbance. If the patient responded, “1. Painful”, there was no pain disturbance. If the subject complained of it being “2. Prickly”, pain disturbance was judged to be absent if pain was felt by pressing harder, and pain disturbance was judged to be present if there was no pain when pressing harder. If the patient complained that it was “3. Not prickly” or there was “4. No sense of touch”, he or she was considered to have a pain disturbance.

The chest and the dorsal hand were then compared by using a locked pain needle. At this point, instead of asking, “Which is stronger?” they were asked, “Are they equal, or is one stronger than the other?”. If the response was slow or ambiguous, they were asked, “Are they almost equal?” or, after stimulating several points, they were asked, “How about on average?”. Areas that were considered depressed were marked with shaded lines in the person-shaped diagram in the medical record.

If the dorsal hand (foot) was weaker than the chest, the chest was compared to the forearm (lower leg). If the forearm (lower leg) was weaker than the chest, the chest and upper arm (thigh) were compared. If the chest and dorsal hand (foot) were equal in degree or the dorsal hand (foot) was stronger, the chest was compared to the fingers of the hand for at least the index, middle, and ring fingers.

The chest and perioral (the bilateral upper and lower sides of the mouth) areas were compared. Comparisons were made between the perioral and buccal areas and between the perioral and forehead areas. When the pain disturbance was considered irregular, the intensity was checked in addition to the above procedure, and a shaded line was marked on the person-shaped diagram in the medical record.

#### 2.3.3. Minimal Tactile Sense

Minimal tactile sense was tested using a set of 20 Semmes–Weinstein monofilaments on the lips, chest, both index fingers, and both thumb toes. Semmes–Weinstein monofilaments are composed of 20 different thicknesses of single nylon fibers and can apply between 0.008 g and 300 g of pressure to the surface of the nylon fibers when bent [11,14,15]. The tactile sensation was tested by placing the tentacles perpendicular to the surface of the skin, with the nylon fibers bent to approximately 90°. The subjects were first told where to stimulate, had their eyes closed, and were instructed to answer “yes” “as soon as you realize that you feel a touch”. At this point, the subjects were instructed to “always answer if you know even a little”; they could make a decision after one trial, but if they were unsure, they were stimulated an odd number of times, and the smallest value that was answered as being felt by the majority of the stimuli was used as the threshold value. The number of grams of the thinnest antennae that could be perceived was used as the threshold value. This method was the same as in previous studies [11,15].

#### 2.3.4. Vibration Sense

The vibration sense test was performed using a C-tuning fork [11,15], which is used for tuning musical instruments, on the chest, the radial side of both wrists, and the lateral parts of both ankles. The tuning fork was struck against the examiner’s knee or other body part with maximum force, and a stopwatch was started as soon as it was struck. The lower part of the tuning fork was applied to each body part, and the examiner instructed, “Say ‘hi’ if you don’t feel these vibrations at all.” When they heard the “hi” response, the stopwatch was stopped, and the number of seconds was noted. This method was the same as in previous studies [11,15].

### 2.4. Method of Analysis

As the purpose of the present analysis was to calculate the prevalence of symptomatic rates in non-designated areas, the analysis was limited to those with a history of residence in the districts during the period 1953–1968, for which there is no disagreement as to the timing of seafood consumption indicating exposure in the designated areas and no history of residence in the designated areas. Therefore, those subjects without a history of residence in the district during the period 1953–1968 or with a history of residence in the designated area were excluded from the analysis and were also excluded from the total population count.

In the case of methylmercury contamination in Minamata and Niigata in Japan, the levels of mercury in the hair and other parts of the body have only been measured in a small portion of the population. Although the most severe period of contamination is considered to have been in the 1950s and 1960s, the actual head-hair mercury values were measured in only 1700 people per year during a very short period of time from 1960–1962 [16,17,18,19]. Therefore, we estimated exposure by region of residence, occupation, and seafood intake.

The subjective symptoms, the degree of touch, pain, and chest pain disturbance upon examination, the minimal tactile sense using Semmes–Weinstein monofilaments, and the threshold of vibratory sensation of the residents of the three districts were compared.

In addition, our previous studies have shown that the frequency of subjective symptoms can be proportional to the degree of exposure and health problems and that the pattern of subjective symptoms can be used as material for qualitative studies on whether or not methylmercury is likely to cause health problems [2,11,15]. Therefore, we calculated the frequency of subjective symptoms and performed a correlation analysis of the pattern of subjective symptoms.

For the touch and pain senses, we calculated the frequencies of peripheral sensory disturbance of four limbs, generalized (trunk and four limbs) sensory disturbance, and perioral sensory disturbance, which are commonly observed in methylmercury poisoning; based on these results, we calculated the attributable fractions. The attributable fractions, which indicate the proportion of symptoms attributable to exposure, are calculated by subtracting the prevalence of symptoms in the unexposed group from the prevalence in the exposed group and dividing by the prevalence in the exposed group [20].

The attributable fractions were calculated based on the following two assumptions about the prevalence of non-surveyed residents in the remote contaminated area under question: 1. the prevalence of non-surveyed residents was assumed to be the same as that of the surveyed residents; 2. all the non-surveyed residents of the remote contaminated area lived in the area between 1953 and 1968, had no history of living in the designated area, and had no sensory disturbances.

Tactile thresholds using the Semmes–Weinstein monofilament and vibratory thresholds were used to estimate the patterns of sensory impairment that might be found in each of these areas and in which parts of the body they were more likely to be impaired.

Statistical calculations were performed using MS Excel and STATA software (version 14).

## 3. Results

### 3.1. Background of Subjects

The data of the subjects examined in each district are presented in Table 1. The top row of the table (A) is the number of births before 1968; the next row (B) is the number of persons examined, and (C) is the number of persons without a history of residence in the district during the period 1953–1968 or with a history of residence in the designated area. Subtracting these numbers (C) from the number of persons screened (B) yields the final number of subjects for analysis (D). The original number of subjects (A) minus (D) is the maximum number (E) of subjects with a history of residence in the area between 1953 and 1968 and without a history of residence in the designated area (the number obtained by dividing (D) by (E) is the minimum proportion (F) of those who have been screened from the population in the area of residence.

In Miyanokawachi District, 108 out of 206 persons were examined, of which 38 persons with no history of residence in the district during the period from 1953–1968 or with a history of residence in the designated area were excluded, leaving 70 persons (male/female = 35/35: age 69.9 ± 10.8 years) for the total sample. The minimum ratio of the surveyed population to the maximum population covered was 70/168 = 41.7%.

In Himedo District, 107 out of 198 persons were examined, of which 18 persons who met the same conditions as those in Miyanokawachi District described above were excluded, leaving 89 persons (male/female = 45/44: age 71.4 ± 11.4 years) for the total sample. The minimum ratio of the surveyed population to the maximum population covered was 89/180 = 49.4%.

In Nagashima District, 71 out of 128 persons were examined, of which 26 persons who met the same conditions as those in Miyanokawachi District described above were excluded, leaving 45 persons (male/female = 29/16: age 68.3 ± 10.3 years) for the total sample. The minimum ratio of the surveyed population to the maximum population covered was 45/102 = 44.1%.

Miyanokawachi and Himedo Districts are located in the Yatsushiro Sea, while Hoppouzaki and Ohama Districts in Nagashima are the outlet of the Yatsushiro Sea and also face the outer world of the East China Sea. The interviews revealed that the fishermen in these areas have fished in both the Yatsushiro Sea and the East China Sea, and local residents have also obtained and eaten fish from both.

In the control area, Amami District, 72 out of 1043 people were examined, and 70 subjects (male/female = 21/49; age: 71.9 ± 9.9 years) were included in the sample after excluding two subjects who had previously lived in the designated area in the past. The minimum ratio of the surveyed population to the maximum population covered was 70/1041 = 6.7%.

There were no differences in drinking history, but current smoking (*p* < 0.05) and smoking history (*p* < 0.01) were significantly lower in the Amami area than in the three remote contaminated areas. The frequency and amount of seafood consumption were significantly higher in Miyanokawachi, Himedo, Nagashima, and Amami, in that order (*p* < 0.01). The number of fishermen employed by themselves and their family members was also significantly higher in Miyanokawachi, Himedo, Nagashima, and Amami, in that order (*p* < 0.01). History of Minamata disease application was significantly higher in Miyanokawachi, Himedo, Nagashima, and Amami, in that order (*p* < 0.05: Miyanokawachi and Himedo; Himedo and Nagashima; *p* < 0.01: other combinations). Family history of Minamata disease was more frequent in Miyanokawachi, Himedo, Nagashima, and Amami, in that order (*p* < 0.01), except for Miyanokawachi and Himedo. There were no significant differences in complications among the four districts.

### 3.2. Complaints

**Figure 2:**
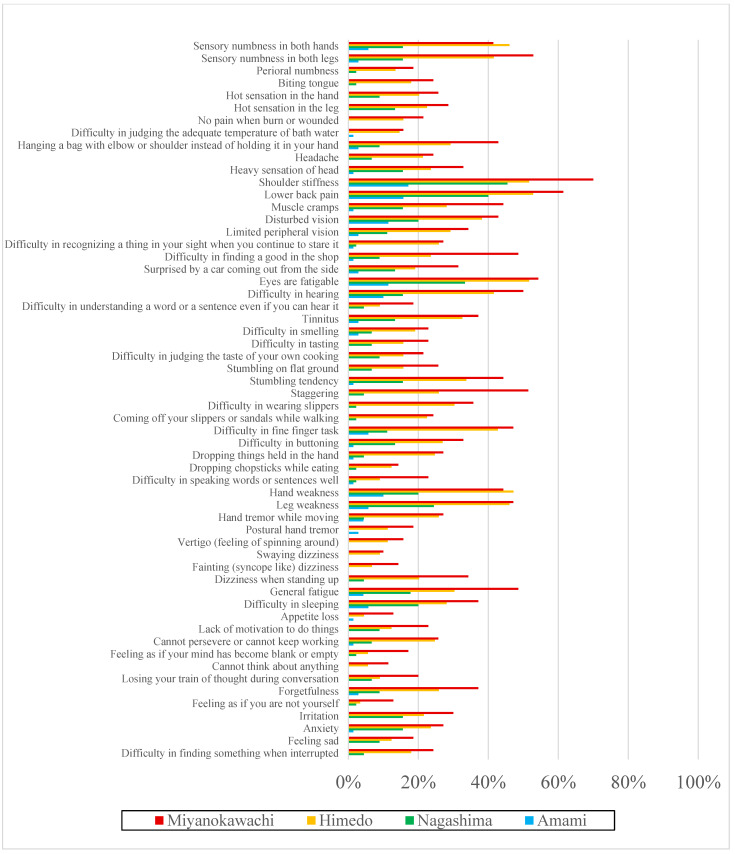
Prevalence of symptoms (Always) in each area (n = 274).

**Figure 3:**
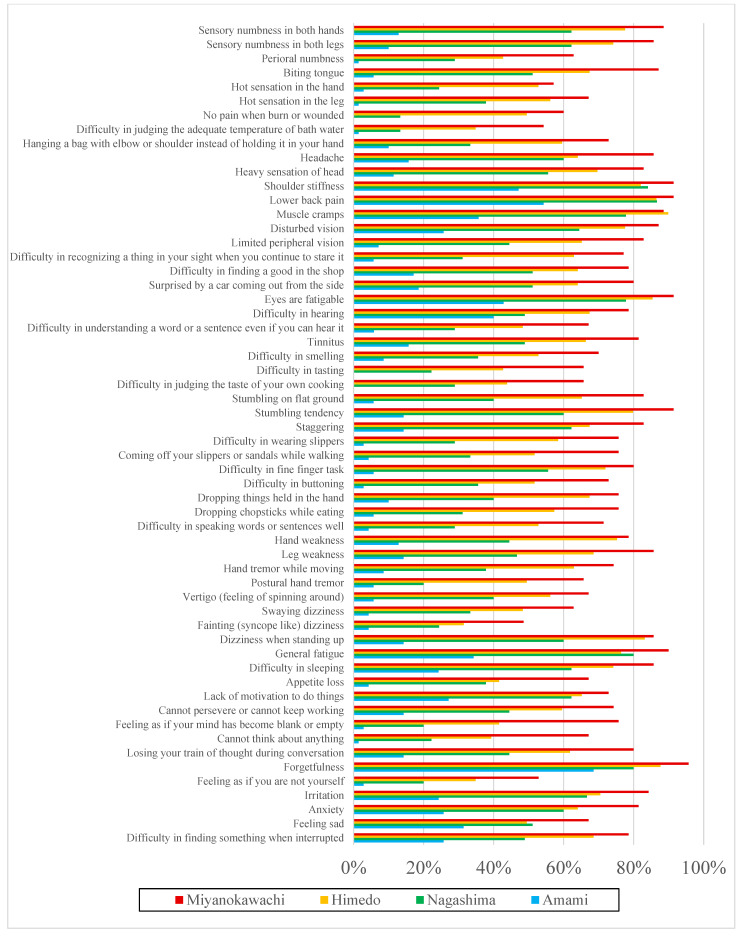
Prevalence of symptoms (Always and Sometimes) in each area (n = 274).

The results of the questionnaire on complaints are presented in Figure 2 and Figure 3 and Tables S1 and S2. In Tables S1 and S2, the odds ratios were calculated for complaints in the three districts and were compared with the controls after adjustment for sex, age, presence of the complication being investigated, and previous drinking history. The proportion of “always” present symptoms was higher in Miyanokawachi, Himedo, Nagashima, and Amami (Figure 2; Table S1), and the proportion of significantly higher symptoms in the three remote contaminated areas compared with controls was 100% (58/58) in Miyanokawachi, 81% (47/58) in Himedo and 28% (16/58) in Nagashima. “Always” or “Sometimes” symptoms were also more frequent in Miyanokawachi, Himedo, Nagashima, and Amami, in that order (Figure 3; Table S2); significantly higher proportions of symptoms in the three remote contaminated areas compared with controls was 100% (58/58) in Miyanokawachi, 100% (58/58) in Himedo, and 98% (57/58) in Nagashima.

Thus, progressively higher rates were found for sensory, motor, and physical pain; general complaints; and psychological and intellectual problems, in proportion to the level of exposure. The fact that the additional rate of “Sometimes” symptoms was higher than the rate of “Always” symptoms only in Nagashima suggests that the symptoms of methylmercury toxicosis are latent.

The similarity of the symptoms of each group was examined by looking at the correlation of the percentage pattern of subjective complaints between the groups (Table 2 and Table 3). Both “Always” and “Always and Sometimes” complaints showed high correlation coefficients of 0.8 or higher between the exposure groups. The correlation coefficients between Amami and Miyanokawachi and between Amami and Himedo were all less than 0.8, while those between Amami and Nagashima were slightly above 0.8. This may be due to the fact that each complaint had a mixture of being more specific and more nonspecific as health effects of methylmercury exposure and that the overall health effects were milder in Nagashima than in Miyanokawachi or Himeido, resulting in a stronger effect of nonspecific symptoms.

The occurrence of muscle cramps in various parts of the body was also higher in Miyanokawachi, Himedo, Nagashima, and Amami, in that order, overall (Table 4). The differences within the remote contaminated areas were small, with significant differences found only in the hands between Miyanokawachi and Himedo and Himedo and Nagashima. Regarding the differences between the three remote contaminated areas and the control area of Amami, significant differences were found for all body parts for Miyanokawachi, hands and feet for Himedo, and only feet for Nagashima.

### 3.3. Sensory Function Tests

#### 3.3.1. Touch and Pain in the Limbs and Trunk and Degree of Chest Pain

**Figure 4:**
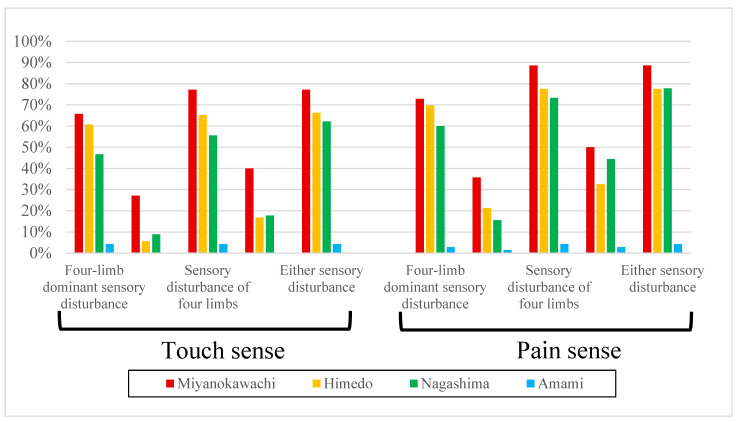
Prevalence of sensory disturbances in each area (1).

**Figure 5:**
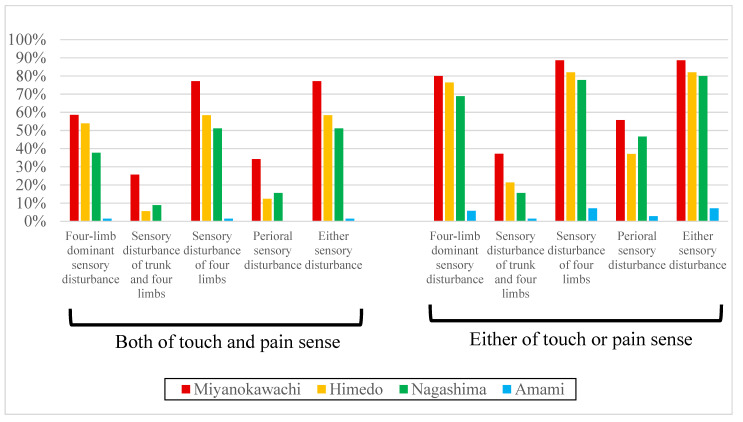
Prevalence of sensory disturbances in each area (2).

**Figure 6:**
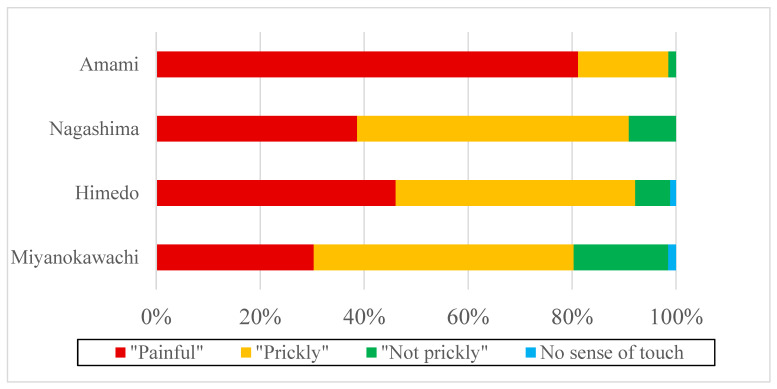
Results of pain sense on the chest.

The results of the touch and pain tests and the degree of thoracic pain perception are shown in Table 5, Figure 4, Figure 5 and Figure 6.

The incidence of four-limb-dominant disturbance, the disturbance of the trunk and four limbs, the disturbance of four limbs, perioral disturbance, and disturbances of any of the above using a brush was generally higher in Miyanokawachi, Himedo, Nagashima, and Amami, in that order. The perioral disturbance was almost equal between Nagashima and Himedo. Compared with the control, Amami, there were significant differences in the incidence of four-limb-dominant disturbance, the disturbance of four limbs, perioral disturbance, and the disturbance of any of the above in the three remote contaminated areas. However, the disturbance of the trunk and four limbs was significantly different only in Amami and Miyanokawachi (Table 5, Figure 4).

The incidence of four-limb-dominant disturbance, the disturbance of the trunk and four limbs, the disturbance of four limbs, perioral disturbance, and disturbances in any of the above using the pain needle was generally higher in Miyanokawachi, Himedo, Nagashima, and Amami, in that order. However, perioral disturbance was slightly more common in Nagashima than in Himedo. Compared with the control, Amami, there were significant differences in the three remote contaminated areas in the incidence of four-limb-dominant disturbance, the disturbance of the trunk and four limbs, the disturbance of four limbs, perioral disturbance, and the disturbance of any of the above (Table 5, Figure 4).

The proportion of those with disturbance of both touch and pain senses was generally higher in Miyanokawachi, Himedo, Nagashima, and Amami, in that order. However, the disturbance of both the limbs and trunk and disturbance around the mouth were slightly more common in Nagashima than in Himedo. The proportion of those with disturbance of either touch or pain senses was generally higher in Miyanoawachi, Himedo, Nagashima, and Amami, in that order. However, perioral disturbance was slightly more common in Nagashima than in Himedo. Compared with the control, Amami, the proportion of those with both (or either) touch and/or pain senses impaired was significantly higher in the three remote contaminated areas (Table 5, Figure 5).

In the chest pain perception tests, significantly fewer people in the three remote contaminated areas “felt pain” compared to those in Amami, and significantly more people in the three areas “felt prickly” compared to those in Amami. Significantly more people in Miyanokawachi than in Amami “had felt touched, but it was not prickly”. There was no difference in the number of respondents who “did not even know they had been touched” in any of the four districts (Table 5; Figure 6).

#### 3.3.2. Minimal Tactile Sense

**Figure 7:**
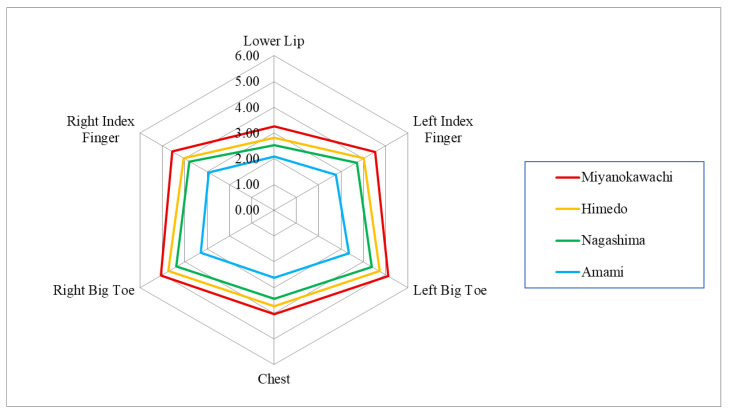
Quantitative minimal tactile sensory measurement by Semmes–Weinstein monofilaments (Unit = Evaluator size: log [g] + 4).

The thresholds of minimal tactile sense at each body site by Semmes–Weinstein monofilaments are shown in Table 6 and Figure 7. The thresholds for the six body sites were all higher in the order of Miyanokawachi, Himeido, Nagashima, and Amami, and all districts showed significant differences among the four districts.

#### 3.3.3. Vibration Sense

**Figure 8:**
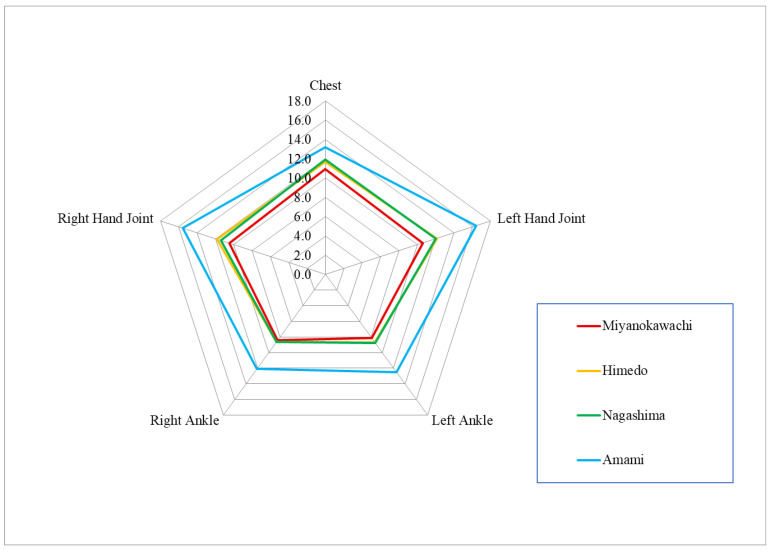
Quantitative vibration sensory measurement using a tuning fork (Unit = second).

The number of seconds in which each body part was able to perceive the vibration sense of the C-tuning fork is shown in Table 7 and Figure 8. All five body sites showed smaller values in the order of Miyanokawachi, Himedo ≈ Nagashima, and Amami. Three remote contaminated sites showed significantly smaller values for all sites compared to the control, Amami. Among the remote contaminated districts, Miyanokawachi showed significantly lower values at both hand joints and a trend toward smaller values at the lips and both foot joints compared to Himedo. Compared with Nagashima, Miyanokawachi showed a trend toward smaller values at all body sites, but no significant differences were observed. Himedo and Nagashima showed almost the same level of disability, with no significant differences.

### 3.4. Attributable Fraction in the Exposed Groups

The attributable fractions in the exposed groups for each type of sensory disturbance in each district, when compared with the control, Amami, are shown in Table 8.

The attributable fractions in the exposed groups of the remote contaminated area as the number of surveyed persons (assuming that the prevalence of the residents of the non-surveyed residents was the same as that of surveyed residents) are shown in the upper part of Table 8. In this case, the attributable fractions in the exposed groups when both touch and pain senses were disturbed ranged from 96.2 to 100%, and the attributable fractions in the exposed groups for each sensory disturbance pattern when either touch or pain senses were disturbed ranged from 90.8 to 96.2%.

Assuming that all persons not surveyed in the remote contaminated area lived in the area between 1953 and 1968, had no history of residence in the designated area, and did not exhibit all patterns of sensory disturbance, the attributable fractions in the exposed groups are shown in the lower part of Table 8. In these cases, the attributable fractions in the exposed groups for each sensory disturbance pattern when both touch and pain senses were disturbed ranged from 91.4 to 100%, and the attributable fractions for each sensory disturbance pattern when either touch or pain senses were disturbed ranged from 79.2 to 90.8%.

## 4. Discussion

In the area surrounding Minamata, few direct exposure indicators of methylmercury, such as total mercury levels in residents’ hair, have been measured in the past. Therefore, we used the amount and frequency of fish and shellfish consumption and the occupational history of individuals and family members as indirect indicators of residents’ exposure to methylmercury. This was a similar approach to what was used in previous studies [11,14,15].

These indirect methylmercury exposure indices were higher in the order of Miyanokawachi, Himeido, Nagashima, and Amami, with proportionally more complaints and sensory disturbances and minimal tactile sense abnormalities in the order of Miyanokawachi, Himeido, Nagashima, and Amami. These adverse health data indicate that health effects due to methylmercury exposure have been observed in more distant locations than previously known. Although Nagashima also consumed fish from the East China Sea and may have had less total methylmercury exposure than the other two districts, the symptom data indicated that health effects were present, albeit milder.

Vibration sense abnormalities were also observed mostly in Miyanokawachi, Himeido ≈ Nagashima, and Amami, in that order, but the differences among the remote contaminated areas were smaller, and the vibration sense test using the C-tuning fork may have been less sensitive to detecting the differences among these three remote contaminated areas compared with the minimal tactile sense using the Semmes–Weinstein monofilament.

Somatosensory disturbances due to chronic methylmercury toxicosis are characterized by damage to the parietal lobes of the cerebrum, which are thought to be the primary responsible lesion, beginning at the tips of the four limbs in milder disease and leading to superficial sensory deficits throughout the body in more severe disease [11,14,15]. On the other hand, sensory disturbances due to peripheral neuropathy, such as polyneuropathies, occur earliest in the lower extremities and then in the upper extremities and are least likely to occur in the trunk and face. The fact that general sensory disturbances—which are rare in the general population—were found in the three remote contaminated areas and that sensory disturbances were found in the lips and chest in the minimal tactile sense and in the chest in the vibratory sense suggests that the sensory disturbances found in the residents of these three remote contaminated areas are related to methylmercury exposure [11,14,15].

In 1999, Tsuda et al. [6] showed that when peripheral sensory disturbances of the four limbs were present in people exposed to methylmercury, the probability of Minamata disease (attributable fractions) became very high (Table 9: Reprinted from [6]). Tatetsu et al. found sensory disturbances in 10.5% (82/784) of the residents in Minamata in 1971–72 [7]. Fujino found sensory disturbance of the four limbs in all cases (100%; 31/31) in the contaminated areas of Katsurajima, Izumi. In a survey of control areas, Fujino et al. found no limb sensory disturbance (0%; 0/33) in Nishiamuro, Amami Island [5], Ninomiya et al. found 0.7% (1/142) in Ichiburi, Miyazaki Prefecture [6], and Kumamoto et al. found 0.2% (3/1270) in rural areas in Kumamoto Prefecture [21]. These results indicate that the attributable fraction in the exposed groups for sensory disturbance of the four limbs in the contaminated area in question was well over 90%.

Although the degree of methylmercury exposure in this study was considered to be relatively low, compared to previous areas [5,6,12,13], the fact that the attributable fraction in the exposed groups in these three remote contaminated areas was at least 79.2% indicates that when the relevant sensory disturbance was observed in these three areas, it cannot be denied that it was caused by methylmercury exposure. These values indicate that this may be an expression of the degree of contamination.

Compared with the control, Amami, the frequency and amount of seafood consumption were higher in the three remote contaminated areas, but the difference in the incidence of diabetes, stroke, and spinal cord disease, which can cause sensory disturbances, among the four areas was not large enough to explain these differences (Table 1), and the difference in the frequency of sensory disorders could not be explained by factors other than methylmercury. Furthermore, these comorbidities do not cause generalized sensory disturbance, and it is also highly unlikely that four-limb-dominant sensory disturbance would occur [27,28]. Therefore, it is also considered highly unlikely that the high incidence of sensory disturbance in the three remote contaminated areas is caused by the effects of spinal disease due to fishing work.

Although the low screening rate in Amami compared to other districts may be regarded as a weakness of this study, considering that the rates of sensory impairment in the general population studied so far have all been low [5,6,21], it is at least unlikely that the bias is in the direction of increasing the risk ratio of the exposed groups.

The fact that the purpose of the screening in the contaminated area was reportedly to diagnose methylmercury poisoning may be considered a factor of bias, but such a bias is impossible to eliminate in principle for contamination-related health problems in such a population, and does not diminish the value of this study.

In 2020, James et al. [29] proposed a theory that the cause of Minamata disease was not methylmercury, but rather α-mercuri-acetaldehyde, based on the fact that in one of the historical cat specimens that had died in 1959 and had been preserved for 60 years, methylmercury was absent, and α-mercuri-acetaldehyde and inorganic mercury were present. However, this showed that α-mercuri-acetaldehyde was one of the factors resulting from exposure, and there is no information other than the one cat that shows a relationship between α-mercuri-acetaldehyde and health problems.

On the other hand, the causal relationship between methylmercury exposure and health problems in Minamata disease is clear. The facts that there was methylmercury contamination in the Chisso factory [30], in fish and shellfish [30,31], in humans (hair, umbilical cord, brain) [1,16,17,18,19,32,33] in the Minamata area, that the condition of the Minamata disease patients [2,5,11,12,14] was the same as that reported by Hunter et al. in the United Kingdom [34] and the cases in Iraq [35], and that there was a clear relationship between hair mercury and symptoms [36,37] are all irrefutable facts [38,39]. The health problems in the remote contaminated areas in this study were also similar (though milder) to those of past Minamata disease patients [1,2,5,11], and it is believed that methylmercury is definitely the primary cause.

Although there is a possibility that α-mercuri-acetaldehyde may be one of the factors causing the health problems of Minamata disease, there is no evidence to suggest that it replaces methylmercury.

## Figures and Tables

**Table 1:** Demographic characteristics of subjects in each area (n = 274).

		Miyanokawachi	Himedo	Nagashima	Amami	Total
		(n = 70)	(n = 89)	(n = 45)	(n = 70)	(n = 274)
(A)	Population (born before December 1968)	206	198	128	1043	1575
(B)	Examined residents	108	107	71	72	358
(C)	Examined residents who had not lived in the district between 1953 and 1968 or who had lived in the designated area	38	18	26	2	84
(D)	Examined residents who had lived in the district between 1953 and 1968 and who had not lived in the designated area: (B)–(C)	70	89	45	70	274
(E)	Maximum population of the district with the same condition of (D)	168	180	102	1041	1491
(F)	Minimum ratio of examined residents/maximum population: (D)/(E)	41.7%	49.4%	44.1%	6.7%	18.4%
Sex, n (%)						
	Male	35 (50.0)	45 (50.6)	29 (64.4)	21 (30.0)	130 (49.1)
	Female	35 (50.0)	44 (49.4)	16 (35.6)	49 (70.0)	144 (54.3)
Age						
	Mean ± SD ^a^	69.9 ± 10.8	71.4 ± 11.4	68.3 ± 10.3	71.9 ± 9.9	70.6 ± 10.7
	Range (min–max)	51–94	48–96	49–94	53–93	48–96
Medial examination rate						
	Examined subjects/population (%)	70/168 (41.7%)	89/180 (49.4%)	45/102 (44.1%)	70/1041 (6.7%)	274/1491 (18.4%)
Smoking, n (%)						
	Nonsmoker (current)	59 (84.3)	76 (85.4)	38 (84.4)	61 (98.4)	234 (88.0)
	Smoker (current)	11 (15.7)	13 (14.6)	7 (15.6)	1 (1.6)	32 (12.0)
	Smoker (including past)	25 (35.7)	28 (31.5)	21 (46.7)	5 (8.1)	79 (29.7)
Alcohol drinking, n (%)						
	Nondrinker (current)	41 (58.6)	62 (69.7)	21 (46.7)	36 (58.1)	160 (60.2)
	Drinker (current)	29 (41.4)	27 (30.3)	24 (53.3)	26 (41.9)	106 (39.8)
	Drinker (including past)	30 (42.9)	33 (37.1)	24 (53.3)	27 (43.5)	114 (42.9)
Frequency of fish intake, n (%)						
	Three times a day	51 (73.9)	38 (43.7)	6 (13.3)	5 (7.1)	100 (36.9)
	Twice a day	9 (13.0)	24 (27.6)	13 (28.9)	6 (8.6)	52 (19.2)
	Once a day	7 (10.1)	13 (14.9)	17 (37.8)	9 (12.9)	46 (17.0)
	More than once a week	1 (1.4)	12 (13.8)	7 (15.6)	42 (60.0)	62 (22.9)
	Less than once a week	1 (1.4)	0 (0.0)	2 (4.4)	8 (11.4)	11 (4.1)
Amount of fish intake, n (%)						
	Five dishes or more a day	15 (21.7)	10 (11.4)	1 (2.3)	0 (0.0)	26 (9.6)
	Three to four dishes a day	28 (40.6)	22 (25)	2 (4.5)	1 (1.4)	53 (19.6)
	Two dishes a day	17 (24.6)	23 (26.1)	18 (40.9)	3 (4.3)	61 (22.6)
	One dish a day	9 (13.0)	32 (36.4)	19 (43.2)	53 (76.8)	113 (41.9)
	Half dishes a day	0 (0.0)	1 (1.1)	4 (9.1)	12 (17.4)	17 (6.3)
Occupation, n (%)						
	Fisherman (subject)	29 (42.0)	26 (29.2)	1 (2.2)	1 (1.4)	57 (21.0)
	Fishery-related occupation (subject)	1 (1.4)	7 (7.9)	1 (2.2)	0 (0.0)	9 (3.3)
	Non-fisherman (subject)	39 (56.5)	56 (62.9)	43 (95.6)	68 (98.6)	206 (75.7)
	Fisherman (subject’s parent)	56 (80.0)	49 (55.1)	16 (35.6)	13 (18.6)	134 (48.9)
	Fishery-related occupation (subject’s parent)	0 (0.0)	8 (9.0)	2 (4.4)	0 (0.0)	10 (3.6)
	Non-fisherman (subject’s parent)	14 (20.0)	32 (36.0)	27 (60.0)	57 (81.4)	130 (47.4)
Complications, n (%)						
	Diabetes mellitus	10 (14.3)	9 (10.1)	4 (8.9)	9 (12.9)	32 (11.7)
	Cerebrovascular diseases	4 (5.7)	0 (0.0)	1 (2.2)	1 (1.4)	6 (2.2)
	Diseases of spinal cord or vertebra	3 (4.3)	4 (4.5)	2 (4.4)	4 (5.7)	13 (4.7)
	Vibration disease	4 (5.7)	1 (1.1)	1 (2.2)	0 (0.0)	6 (2.2)
	Other neurological diseases	5 (7.1)	2 (2.2)	3 (6.7)	3 (4.3)	13 (4.7)
	Either disease listed above	17 (24.3)	15 (16.9)	11 (24.4)	15 (21.4)	58 (21.2)
Family history of application for Minamata disease, n (%)		57 (81.4)	57 (64.0)	18 (40.0)	0 (0.0)	132 (48.2)
Family history of Minamata disease, n (%)		54 (77.1)	55 (61.8)	11 (24.4)	0 (0.0)	120 (43.8)

^a^: *p* < 0.05 (Nagashima vs. Amami); n.s. (all other combinations).

**Table 2:** Correlation of prevalence of symptoms (Always) among each area.

Correlation/(*p*-Value)	Miyanokawachi	Himedo	Nagashima
Himedo	0.9063		
	(0.0000)		
Nagashima	0.8157	0.8137	
	(0.0000)	(0.0000)	
Amami	0.7397	0.7970	0.8383
	(0.0000)	(0.0000)	(0.0000)

**Table 3:** Correlation of prevalence of symptoms (Always and Sometimes) among each area.

Correlation/(*p*-Value)	Miyanokawachi	Himedo	Nagashima
Himedo	0.9069		
	(0.0000)		
Nagashima	0.8444	0.8809	
	(0.0000)	(0.0000)	
Amami	0.6401	0.7055	0.8270
	(0.0000)	(0.0000)	(0.0000)

**Table 4:** Prevalence of muscle camps in each area.

	Miyanokawachi	Himedo	Nagashima	Amami
Feet	61 (87%)	78 (87%)	36 (81%)	33 (47%)
Hands	45 (64%)	49 (55%)	14 (31%)	2 (2%)
Chest–Abdomen	9 (12%)	6 (6%)	2 (4%)	0 (0%)
Face–Head	8 (11%)	3 (3%)	4 (9%)	1 (1%)
Experience	65 (92%)	83 (93%)	36 (81%)	34 (47%)
Total	70	89	44	70

**Table 5:** Prevalence of sensory disturbances in each area and adjusted * odds ratios (OR) for the association between area and sensory disturbances (n = 274).

		Miyanokawachi	Himedo	Nagashima	Amami
Touch disturbance					
	Four-limb-dominant sensory disturbance				
		46/70 (65.7)	54/89 (60.7)	21/45 (46.7)	3/70 (4.3)
		43 (12–155)	36 (10–128)	18 (4.8–69)	1 (reference)
	Sensory disturbance of trunk and four limbs **				
		19/70 (27.1)	5/89 (5.6)	4/45 (8.9)	0/70 (0.0)
		32 (3.9–261)	2.7 (0.3–25)	8.1 (0.8–80)	1 (reference)
	Sensory disturbance of four limbs				
		54/70 (77.1)	58/89 (65.2)	25/45 (55.6)	3/70 (4.3)
		77 (21–285)	42 (12–149)	27 (7.1–103)	1 (reference)
	Perioral sensory disturbance **				
		28/70 (40.0)	15/89 (16.9)	8/45 (17.8)	0/70 (0.0)
		43 (5.6–331)	12 (1.5–94)	17 (2–139)	1 (reference)
	Either sensory disturbance				
		54/70 (77.1)	59/89 (66.3)	28/45 (62.2)	3/70 (4.3)
		74 (20–276)	43 (12–153)	29 (7.6–110)	1 (reference)
Pain disturbance					
	Four-limb-dominant sensory disturbance				
		51/70 (72.9)	62/89 (69.7)	27/45 (60)	2/70 (2.9)
		108 (23–509)	104 (22–486)	61 (12.6–298)	1 (reference)
	Sensory disturbance of trunk and four limbs				
		25/70 (35.7)	19/89 (21.3)	7/45 (15.6)	1/70 (1.4)
		35 (4.5–269)	14 (1.8–111)	11 (1.3–98)	1 (reference)
	Sensory disturbance of four limbs				
		62/70 (88.6)	69/89 (77.5)	33/45 (73.3)	3/70 (4.3)
		187 (45–773)	87 (23–326)	70 (17–282)	1 (reference)
	Perioral sensory disturbance				
		35/70 (50.0)	29/89 (32.6)	20/45 (44.4)	2/70 (2.9)
		32 (7.1–142)	15 (3.3–65)	24 (5.2–115)	1 (reference)
	Either sensory disturbance				
		62/70 (88.6)	69/89 (77.5)	35/45 (77.8)	3/70 (4.3)
		198 (48–824)	93 (24–350)	84 (20–345)	1 (reference)
Both of touch and pain disturbance					
	Four-limb-dominant sensory disturbance				
		41/70 (58.6)	48/89 (53.9)	17/45 (37.8)	1/70 (1.4)
		92 (12–708)	79 (10–604)	39 (4.9–316)	1 (reference)
	Sensory disturbance of trunk and four limbs **				
		18/70 (25.7)	5/89 (5.6)	4/45 (8.9)	0/70 (0.0)
		31 (3.7–252)	2.6 (0.3–24)	8.0 (0.8–79)	1 (reference)
	Sensory disturbance of four limbs				
		54/70 (77.1)	52/89 (58.4)	23/45 (51.1)	1/70 (1.4)
		225 (28–1771)	106 (14–809)	73 (9.1–582)	1 (reference)
	Perioral sensory disturbance **				
		24/70 (34.3)	11/89 (12.4)	7/45 (15.6)	0/70 (0.0)
		34 (4.4–263)	8.2 (1.02–66)	13 (1.5–108)	1 (reference)
	Either sensory disturbance				
		54/70 (77.1)	52/89 (58.4)	23/45 (51.1)	1/70 (1.4)
		225 (28–1771)	106 (14–809)	73 (9.1–582)	1 (reference)
Either of touch or pain disturbance					
	Four-limb-dominant sensory disturbance				
		56/70 (80.0)	68/89 (76.4)	31/45 (68.9)	4/70 (5.7)
		95 (27–336)	90 (26–314)	50 (14–181)	1 (reference)
	Sensory disturbance of trunk and four limbs				
		26/70 (37.1)	19/89 (21.3)	7/45 (15.6)	1/70 (1.4)
		126 (36–442)	74 (23–237)	54 (15–186)	1 (reference)
	Sensory disturbance of four limbs				
		62/70 (88.6)	73/89 (82)	35/45 (77.8)	5/70 (7.1)
		37 (4.8–286)	14 (1.9–112)	11 (1.3–99)	1 (reference)
	Perioral sensory disturbance				
		39/70 (55.7)	33/89 (37.1)	21/45 (46.7)	2/70 (2.9)
		40 (8.9–178)	18 (4–78)	29 (6.2–135)	1 (reference)
	Either sensory disturbance				
		62/70 (88.6)	73/89 (82.0)	36/45 (80.0)	5/70 (7.1)
		130 (37–460)	83 (25–272)	72 (20–264)	1 (reference)
Sensation by 20 g pain needle on chest					
	“Painful”				
		20/66 (30.3)	41/89 (46.1)	17/44 (38.6)	56/69 (81.2)
		0.12 (0.05–0.26)	0.25 (0.12–0.53)	0.19 (0.08–0.45)	1 (reference)
	“Tickling”				
		33/66 (50.0)	41/89 (46.1)	23/44 (52.3)	12/69 (17.4)
		8.6 (3.8–20)	4.4 (2–10)	5.5 (2.2–14)	1 (reference)
	No “Tickling”, but with touch feeling				
		12/66 (18.2)	6/89 (6.7)	4/44 (9.1)	1/69 (1.4)
		17 (2.1–140)	4.6 (0.5–40)	7.8 (0.8–75)	1 (reference)
	No touch feelings **				
		1/66 (1.5)	1/89 (1.1)	0/44 (0.0)	0/69 (0.0)
		1 (0.1–22)	0.3 (0.0–6.3)	2.1 (0.1–48)	1 (reference)

* Adjusted for age, sex, either complication, and drinking history (including past); ** When prevalence of either district was zero, we postulated that a positive finding was found in the eldest subject in the district and calculated the OR and 95% confidence interval.

**Table 6:** Quantitative minimal tactile sensory measurement by Semmes–Weinstein monofilaments [Average ± SD (n)].

	Lower Lip	Chest	Right Index Finger	Left Index Finger	Right Big Toe	Left Big Toe
Miyanokawachi	3.27 ± 1.13 (70)	4.03 ± 1.00 (70)	4.55 ± 1.00 (70)	4.52 ± 1.07 (70)	5.07 ± 1.01 (70)	5.12 ± 1.00 (70)
Himedo	2.82 ± 0.74 (89)	3.73 ± 0.84 (89)	4.06 ± 0.85 (89)	4.02 ± 0.89 (89)	4.74 ± 0.87 (89)	4.74 ± 0.86 (89)
Nagashima	2.53 ± 0.59 (45)	3.45 ± 0.77 (45)	3.79 ± 0.58 (45)	3.70 ± 0.65 (44)	4.37 ± 0.64 (44)	4.39 ± 0.73 (45)
Amami	2.08 ± 0.30 (70)	2.63 ± 0.70 (70)	2.92 ± 0.58 (70)	2.77 ± 0.56 (70)	3.29 ± 0.83 (70)	3.34 ± 0.76 (70)

Miyanokawachi vs. Himedo: *p* < 0.05: chest, right index finger, *p* < 0.01: all others. Miyanokawachi vs. Nagashima: *p* < 0.01: all. Miyanokawachi vs. Amami: *p* < 0.01: all. Himedo vs. Nagashima: *p* < 0.05: chest, right index finger, left index finger, *p* < 0.01: all others. Himedo vs. Amami: *p* < 0.01: all, Nagashima vs. Amami: *p* < 0.01: all.

**Table 7:** Quantitative vibration sensory measurement using a tuning fork [Average ± SD (n)].

	Chest	Right Hand Joint	Left Hand Joint	Right Ankle	Left Ankle
Miyanokawachi	10.9 ± 4.5 (68)	10.5 ± 5.2 (68)	10.6 ± 5.5 (68)	8.4 ± 4.7 (65)	8.1 ± 4.7 (66)
Himedo	11.7 ± 3.6 (89)	11.8 ± 4.2 (89)	12.1 ± 4.8 (89)	8.6 ± 4.4 (88)	8.7 ± 4.3 (88)
Nagashima	11.9 ± 2.9 (45)	11.4 ± 3.8 (45)	12.0 ± 3.8 (45)	8.7 ± 2.6 (45)	8.8 ± 3.4 (45)
Amami	13.2 ± 2.9 (70)	15.6 ± 3.1 (70)	16.4 ± 3.3 (70)	12.1 ± 3.5 (70)	12.5 ± 4.0 (70)

Miyanokawachi vs. Himedo: *p* < 0.05: chest, right index finger, n.s.: all others. Miyanokawachi vs. Nagashima: n.s.: all. Miyanokawachi vs. Amami: *p* < 0.01: all. Himedo vs. Nagashima: n.s.: all. Himedo vs. Amami: *p* < 0.01: all, Nagashima vs. Amami: *p* < 0.05: lower lip, *p* < 0.01: all others.

**Table 8:** Attributable fraction calculated using the prevalence of touch and pain sense.

			Miyanokawachi	Himedo	Nagashima
Attributable fraction calculated by prevalence from subjects examined actually					
	Both touch and pain sense				
		Four-limb-dominant sensory disturbance	97.6%	97.4%	96.2%
		Sensory disturbance of the trunk and four limbs	100.0%	100.0%	100.0%
		Sensory disturbance of four limbs	98.1%	97.6%	97.2%
		Perioral sensory disturbance	100.0%	100.0%	100.0%
		Either sensory disturbance	98.1%	97.6%	97.2%
	Either of touch or pain sense				
		Four-limb-dominant sensory disturbance	92.9%	92.5%	91.7%
		Sensory disturbance of the trunk and four limbs	96.2%	93.3%	90.8%
		Sensory disturbance of four limbs	91.9%	91.3%	90.8%
		Perioral sensory disturbance	94.9%	92.3%	93.9%
		Either sensory disturbance	91.9%	91.3%	91.1%
Attributable fraction calculated by prevalence from the whole concerned population					
	Both touch and pain sense				
		Four-limb-dominant sensory disturbance	94.1%	94.6%	91.4%
		Sensory disturbance of the trunk and four limbs	100.0%	100.0%	100.0%
		Sensory disturbance of four limbs	95.6%	95.1%	93.7%
		Perioral sensory disturbance	100.0%	100.0%	100.0%
		Either sensory disturbance	95.6%	95.1%	93.7%
	Either of touch or pain sense				
		Four-limb-dominant sensory disturbance	82.9%	84.9%	81.2%
		Sensory disturbance of the trunk and four limbs	90.8%	86.5%	79.2%
		Sensory disturbance of four limbs	80.6%	82.4%	79.2%
		Perioral sensory disturbance	87.7%	84.4%	86.1%
		Either sensory disturbance	80.6%	82.4%	79.8%

**Table 9:** Prevalence of peripheral sensory disturbance of the four limbs in methylmercury-exposed and non-exposed groups and the corresponding attributable fractions (excluding those with symptoms such as visual field constriction or ataxia in addition to sensory disturbance of the four limbs).

No. Principal Investigator (Year of Investigation: Year of Publication: Bibliography No.)	Area	Subjects	Extraction Rate or Screening Rate	Percentage of Peripheral Sensory Disturbance of the Four Limbs in the High Exposure Group	Percentage of Peripheral Sensory Disturbance of the Four Limbs in the Medium Exposure Group	Percentage of Peripheral Sensory Disturbance of the Four Limbs in the Non-Exposed Group	Relative Risk †† [95% Confidence Interval] (Percent of Attributable Fraction)
① Tatetsu (1971: 1972: [7])	Minamata City	Resident survey	82.9% (928/1120)	10.5% (82/784)			49.3 [15–196] (98.0%)
② Tatetsu (1971: 1972: [7])	Goshonoura	Resident survey	93.4% (1723/1845)		2.3% (38/1684)		9.8 [2.9–39.6] (89.7%)
③ Tatetsu (1971: 1972: [7])	ex-Ariake Town	Resident survey	77.6% (904/1165)		2.4% (22/899)		10.6 [3.0–45] (90.6%)
④ Fujino (1975: 1977: [22,23])	Katsurajima	Resident survey	57.2% (127/222)	† 36.7% (18/49) 53.2% (33/62) for noncertified			245.2 [63.5–1109.8] (99.6%)
⑤ Fujino (1976: 1977: [5])	Kakeroma Island, Amami Islands	Resident survey, 30 years and older	47.0% (55/117)			† 0% (0/55)	
⑥ Harada (1976: 1983: [24])	Fukuura, Tsunagi Town	Resident survey, 15 years and older	84.1% (1)	† 52.8% (47/89) 66.7% (84/126) for noncertified			472.6 [133.9–1987.9] (99.8%)
⑦ Harada (1976: 1983: [24])	Yunokuchi, Shishijima	Resident survey, 15 years old and older	100%	† 30.6% (11/36) 43.2% (19/44) for noncertified			185.8 [44.1–901.9] (99.5%)
⑧ Ninomiya (1975–1979: 1995: [14])	Oura, Goshonoura	Resident survey, 20 years and older	25.1% (109/435)		† 37.1% (26/70)		249.6 [68.4–1078.7] (99.6%)
⑨ Ninomiya (1989: 1995: [14])	Ichiburi, Miyazaki Prefecture	Resident survey, 20 years and older	21.5% (142/660)			† 0.7% (1/142)	
⑩ Tokuomi (unknown: 1976: [25])	Mountainous area of Kumamoto Prefecture (Taraki Town, Aso Town, Mifune Town)	General elderly, 50 years and older (not examined group)	unknown			1.1% (1/91)	
⑪ Tokuomi (unknown: 1976: [25])	Kumamoto City	Old people’s home	unknown			0% (0/22)	
⑫ Kumamoto (1989–1991: 1993: [21])	Rural district, Kumamoto Prefecture	Resident survey, 60 years and older at home	83% (1270/1530)			0.2% (3/1270)	
⑬ Osame (1991: 1993: [26])	Remote island K town, Kagoshima Prefecture	Old people, 60 years and older	22.8% (421/1850)			1% (Unknown)	

†: From the original data. All other data are deduced from the literature. ††: Values that are calculated when the data of Kumamoto et al. [21] are used as those with the nonexposed group. Figures in [ ] show 95% confidence intervals for relative risk.

## Data Availability

The data are unavailable due to privacy and ethical restrictions.

## Supplementary Materials

The following supporting information can be downloaded at: https://www.mdpi.com/article/10.3390/toxics12100751/s1, Table S1. Prevalence of symptoms (Always) and adjusted* odds ratios (OR) for the association between area and symptoms (n = 274). Table S2. Prevalence of symptoms (Always and Sometimes) and adjusted* odds ratios (OR) for the association between area and symptoms (n = 274).
