# Supplementary material for: Health Effects of Methylmercury in Coastal Areas of the Yatsushiro Sea, Far from Minamata
Source: Toxics. 2024 Oct 16;12(10):751. doi: 10.3390/toxics12100751 (PMC11511511; doi:10.3390/toxics12100751)
Supplement: Supplementary file 1 [file toxics-12-00751-s001.zip › toxics-3187841-supplementary.pdf]

Table S1. Prevalence of symptoms (Always) and adjusted* odds ratios (OR) for the association between area and symptoms (n=274).

	Miyanokawachi	Himedo	Nagashima	Amami
1	Sensory numbness in both hands			
	29/70 (41.4)	41/89 (46.1)	7/45 (15.6)	4/70 (5.7)
	17 (4.6 - 60)	20 (5.8 - 71)	4.4 (1.04 - 18)	1 (reference)
2	Sensory numbness in both legs			
	37/70 (52.9)	37/89 (41.6)	7/45 (15.6)	2/70 (2.9)
	42 (9.2 - 190)	25 (5.6 - 113)	6.4 (1.2 - 33)	1 (reference)
3	Perioral numbness**			
	13/70 (18.6)	12/89 (13.5)	1/45 (2.2)	0/70 (0)
	17 (2.1 - 135)	11 (1.3 - 86)	1.8 (0.1 - 31)	1 (reference)
4	Biting tongue**			
	17/70 (24.3)	16/89 (18)	1/45 (2.2)	0/70 (0)
	21 (2.7 - 166)	15 (1.9 - 116)	1.5 (0.1 - 25)	1 (reference)
5	Hot sensation in the hand**			
	18/70 (25.7)	18/89 (20.2)	4/45 (8.9)	0/70 (0)
	26 (3.3 - 205)	19 (2.5 - 152)	7.7 (0.8 - 73)	1 (reference)
6	Hot sensation in the leg**			
	20/70 (28.6)	20/89 (22.5)	6/45 (13.3)	0/70 (0)
	34 (4.3 - 269)	25 (3.1 - 193)	14.2 (1.6 - 126)	1 (reference)
7	No pain when burn or wounded**			
	15/70 (21.4)	14/89 (15.7)	0/45 (0)	0/70 (0)
	21 (2.6 - 170)	11 (1.4 - 92)	1.6 (0.1 - 28)	1 (reference)
8	Difficulty in judging the adequate temperature of bath water**			
	11/70 (15.7)	13/89 (14.6)	0/45 (0)	1/70 (1.4)
	13 (1.5 - 102)	12 (1.5 - 97)	1.5 (0.1 - 25)	1 (reference)
9	Hanging a bag with elbow or shoulder instead of holding it in your hand			
	30/70 (42.9)	26/89 (29.2)	4/45 (8.9)	2/70 (2.9)
	29 (6.3 - 130)	15 (3.4 - 69)	3.7 (0.6 - 22)	1 (reference)
10	Headache**			
	17/70 (24.3)	19/89 (21.3)	3/45 (6.7)	0/70 (0)
	23 (2.9 - 178)	17 (2.2 - 134)	5.7 (0.6 - 58)	1 (reference)
11	Heavy sensation of head			
	23/70 (32.9)	21/89 (23.6)	7/45 (15.6)	1/70 (1.4)
	31 (4 - 237)	19 (2.5 - 148)	12 (1.4 - 102)	1 (reference)
12	Shoulder stiffness			
	49/70 (70)	46/89 (51.7)	20/44 (45.5)	12/70 (17.1)
	19 (7.4 - 48)	8.7 (3.6 - 21)	7.2 (2.7 - 19)	1 (reference)
13	Lower back pain			
	43/70 (61.4)	47/89 (52.8)	18/45 (40)	11/70 (15.7)
	9.1 (3.9 - 21)	6.5 (2.8 - 15)	3.7 (1.4 - 9)	1 (reference)

14	Muscle cramps				
		31/70 (44.3)	25/89 (28.1)	7/45 (15.6)	1/70 (1.4)
		53 (6.9 - 407)	24 (3.1 - 181)	13 (1.5 - 111)	1 (reference)
15	Disturbed vision				
		30/70 (42.9)	34/89 (38.2)	9/45 (20)	8/70 (11.4)
		5.9 (2.3 - 15)	4.5 (1.8 - 11)	1.9 (0.6 - 5.7)	1 (reference)
16	Limited peripheral vision				
		24/70 (34.3)	26/89 (29.2)	5/45 (11.1)	2/70 (2.9)
		16 (3.6 - 73)	12 (2.8 - 55)	3.7 (0.7 - 21)	1 (reference)
17	Difficulty in recognizing a thing in your sight when you continue to stare it				
		19/70 (27.1)	23/89 (25.8)	1/45 (2.2)	1/70 (1.4)
		26 (3.3 - 204)	22 (2.8 - 173)	1.5 (0.1 - 26)	1 (reference)
18	Difficulty in finding a good in the shop				
		34/70 (48.6)	21/89 (23.6)	4/45 (8.9)	1/70 (1.4)
		80 (10 - 629)	22 (2.8 - 172)	7.6 (0.8 - 72)	1 (reference)
19	Surprised by a car coming out from the side				
		22/70 (31.4)	17/89 (19.1)	6/45 (13.3)	2/70 (2.9)
		16 (3.5 - 72)	7.5 (1.6 - 35)	5.4 (1.0 - 29)	1 (reference)
20	Eyes are fatigable				
		38/70 (54.3)	46/89 (51.7)	15/45 (33.3)	8/70 (11.4)
		9.0 (3.5 - 23)	7.9 (3.2 - 19)	3.7 (1.3 - 10)	1 (reference)
21	Difficulty in hearing				
		35/70 (50)	37/89 (41.6)	7/45 (15.6)	7/70 (10)
		10 (3.6 - 27)	6.3 (2.4 - 17)	1.6 (0.5 - 5.2)	1 (reference)
22	Difficulty in understanding a word or a sentence even if you can hear it**				
		13/70 (18.6)	8/89 (9)	2/45 (4.4)	0/69 (0)
		17 (2.1 - 134)	6.3 (0.7 - 53)	3.7 (0.3 - 44)	1 (reference)
23	Tinnitus				
		26/70 (37.1)	29/89 (32.6)	6/45 (13.3)	2/70 (2.9)
		17 (3.7 - 74)	13 (3.0 - 59)	4.1 (0.8 - 22)	1 (reference)
24	Difficulty in smelling				
		16/70 (22.9)	17/89 (19.1)	3/45 (6.7)	2/70 (2.9)
		10 (2.1 - 45)	6.9 (1.5 - 32)	2.5 (0.4 - 16)	1 (reference)
25	Difficulty in tasting**				
		16/70 (22.9)	14/89 (15.7)	3/45 (6.7)	0/70 (0)
		19 (2.4 - 151)	12 (1.6 - 98)	4.6 (0.5 - 46)	1 (reference)
26	Difficulty in judging the taste of your own cooking**				
		15/70 (21.4)	14/89 (15.7)	4/45 (8.9)	0/70 (0)
		17 (2.1 - 131)	12 (1.5 - 91)	5.8 (0.6 - 55)	1 (reference)
27	Stumbling on flat ground**				
		18/70 (25.7)	14/89 (15.7)	3/45 (6.7)	0/70 (0)
		30 (3.6 - 242)	11 (1.4 - 92)	6.3 (0.6 - 66)	1 (reference)
28	Stumbling tendency				

		31/70 (44.3)	30/89 (33.7)	7/45 (15.6)	1/70 (1.4)
		63 (8.0 - 488)	36 (4.7 - 281)	14 (1.6 - 122)	1 (reference)
29	Staggering**				
		36/70 (51.4)	23/89 (25.8)	2/45 (4.4)	0/70 (0)
		95 (12 - 759)	23 (2.9 - 180)	3.4 (0.3 - 39)	1 (reference)
30	Difficulty in wearing slippers**				
		25/70 (35.7)	27/89 (30.3)	1/45 (2.2)	0/70 (0)
		56 (6.9 - 449)	38 (4.8 - 305)	1.9 (0.1 - 33)	1 (reference)
31	Coming off your slippers or sandals while walking**				
		17/70 (24.3)	20/89 (22.5)	1/45 (2.2)	0/70 (0)
		27 (3.3 - 216)	23 (2.8 - 182)	1.9 (0.1 - 32)	1 (reference)
32	Difficulty in fine finger task				
		33/70 (47.1)	38/89 (42.7)	5/45 (11.1)	4/70 (5.7)
		22 (6.2 - 82)	17 (4.9 - 62)	2.8 (0.6 - 13)	1 (reference)
33	Difficulty in buttoning				
		23/70 (32.9)	24/89 (27)	6/45 (13.3)	1/70 (1.4)
		43 (5.4 - 345)	31 (3.9 - 243)	12 (1.3 - 108)	1 (reference)
34	Dropping things held in the hand				
		19/70 (27.1)	22/89 (24.7)	2/45 (4.4)	1/70 (1.4)
		28 (3.5 - 220)	23 (2.9 - 178)	3.6 (0.3 - 42)	1 (reference)
35	Dropping chopsticks while eating**				
		10/70 (14.3)	11/89 (12.4)	1/45 (2.2)	0/70 (0)
		12 (1.4 - 101)	7.8 (0.9 - 65)	1.9 (0.1 - 32)	1 (reference)
36	Difficulty in speaking words or sentences well				
		16/70 (22.9)	8/89 (9)	1/45 (2.2)	1/70 (1.4)
		17 (2.1 - 131)	5.1 (0.6 - 43)	1.2 (0.1 - 20)	1 (reference)
37	Hand weakness				
		31/70 (44.3)	42/89 (47.2)	9/45 (20)	7/70 (10)
		8.5 (3.2 - 23)	9.2 (3.5 - 24)	2.7 (0.9 - 9)	1 (reference)
38	Leg weakness				
		33/70 (47.1)	41/89 (46.1)	11/45 (24.4)	4/70 (5.7)
		23 (6.3 - 83)	21 (5.9 - 74)	8.3 (2.1 - 33)	1 (reference)
39	Hand tremor while moving				
		19/70 (27.1)	23/89 (25.8)	2/45 (4.4)	3/70 (4.3)
		13 (2.7 - 58)	10 (2.1 - 44)	1.5 (0.2 - 12)	1 (reference)
40	Postural hand tremor**				
		13/70 (18.6)	10/89 (11.2)	0/45 (0)	2/70 (2.9)
		17 (2.1 - 139)	8.4 (1.01 - 70)	1.6 (0.1 - 28)	1 (reference)
41	Vertigo (feeling of spinning around)**				
		11/70 (15.7)	10/89 (11.2)	0/45 (0)	0/70 (0)
		12 (1.5 - 96)	7.7 (0.9 - 63)	1.5 (0.1 - 25)	1 (reference)
42	Swaying dizziness**				
		7/70 (10)	8/89 (9)	0/45 (0)	0/70 (0)

		7.6 (0.9 - 67)	6.1 (0.7 - 53)	1.7 (0.1 - 30)	1 (reference)
43	Fainting (syncope like) dizziness**				
		10/70 (14.3)	6/89 (6.7)	0/45 (0)	0/70 (0)
		12 (1.4 - 97)	4 (0.5 - 38)	1.7 (0.1 - 29)	1 (reference)
44	Dizziness when standing up**				
		24/70 (34.3)	18/89 (20.2)	2/45 (4.4)	0/70 (0)
		32 (4.1 - 246)	15 (1.9 - 117)	2.9 (0.2 - 33)	1 (reference)
45	General fatigue				
		34/70 (48.6)	27/89 (30.3)	8/45 (17.8)	3/70 (4.3)
		28 (6.2 - 123)	12 (2.7 - 54)	6.2 (1.2 - 31)	1 (reference)
46	Difficulty in sleeping				
		26/70 (37.1)	25/89 (28.1)	9/45 (20)	4/70 (5.7)
		9.4 (3.0 - 30)	6.2 (2.0 - 19)	4.1 (1.1 - 15)	1 (reference)
47	Appetite loss**				
		9/70 (12.9)	4/89 (4.5)	0/45 (0)	1/70 (1.4)
		8.8 (1 - 74)	2.4 (0.3 - 23)	1.3 (0.1 - 22)	1 (reference)
48	Lack of motivation to do things**				
		16/70 (22.9)	11/89 (12.4)	4/45 (8.9)	0/70 (0)
		19 (2.4 - 152)	8.8 (1.1 - 71)	6.4 (0.7 - 61)	1 (reference)
49	Cannot persevere or cannot keep working				
		18/70 (25.7)	22/89 (24.7)	3/45 (6.7)	1/70 (1.4)
		26 (3.3 - 206)	24 (3.1 - 191)	5.4 (0.5 - 55)	1 (reference)
50	Feeling as if your mind has become blank or empty**				
		12/70 (17.1)	5/89 (5.6)	1/45 (2.2)	0/70 (0)
		14 (1.7 - 113)	4 (0.4 - 32)	1.6 (0.1 - 27)	1 (reference)
51	Cannot think about anything**				
		8/70 (11.4)	5/89 (5.6)	0/45 (0)	0/70 (0)
		10 (1.1 - 82)	3 (0.3 - 28)	1.7 (0.1 - 29)	1 (reference)
52	Losing your train of thought during conversation**				
		14/70 (20)	8/89 (9)	3/45 (6.7)	0/70 (0)
		17 (2.1 - 135)	5.5 (0.7 - 46)	5.3 (0.5 - 54)	1 (reference)
53	Forgetfulness				
		26/70 (37.1)	23/89 (25.8)	4/45 (8.9)	2/70 (2.9)
		20 (4.4 - 93)	9.5 (2.1 - 43)	3.0 (0.5 - 18)	1 (reference)
54	Feeling as if you are not yourself**				
		9/70 (12.9)	3/89 (3.4)	1/45 (2.2)	0/70 (0)
		10 (1.2 - 83)	2 (0.2 - 21)	1.8 (0.1 - 30)	1 (reference)
55	Irritation**				
		21/70 (30)	19/88 (21.6)	7/45 (15.6)	0/70 (0)
		25 (3.3 - 197)	16 (2.1 - 126)	11 (1.3 - 94)	1 (reference)
56	Anxiety				
		19/70 (27.1)	21/89 (23.6)	7/45 (15.6)	1/70 (1.4)
		12 (2.5 - 54)	8.9 (2.0 - 41)	5.9 (1.1 - 31)	1 (reference)

57	Feeling sad**			
	13/70 (18.6)	11/89 (12.4)	4/45 (8.9)	0/70 (0)
	16 (2.0 - 129)	8.7 (1.1 - 71)	7.4 (0.8 - 71)	1 (reference)
58	Difficulty in finding something when interrupted**			
	17/70 (24.3)	16/89 (18)	2/45 (4.4)	0/70 (0)
	22 (2.8 - 177)	14 (1.8 - 109)	3.4 (0.3 - 40)	1 (reference)

* Adjusted for age, sex, either complication, and drinking history (including past); ** When prevalence of either district was zero, we postulated that a positive finding was found in the eldest subject in the district and calculated the OR and 95% confidence interval.

Table S2. Prevalence of symptoms (Always and Sometimes) and adjusted* odds ratios (OR) for the association between area and symptoms (n=274).

	Miyanokawachi	Himedo	Nagashima	Amami
1	Sensory numbness in both hands			
	62/70 (88.6)	69/89 (77.5)	28/45 (62.2)	9/70 (12.9)
	67 (22 - 200)	29 (11 - 76)	15 (5.4 - 42)	1 (reference)
2	Sensory numbness in both legs			
	60/70 (85.7)	66/89 (74.2)	28/45 (62.2)	7/70 (10)
	82 (25 - 265)	39 (13 - 114)	21 (6.8 - 66)	1 (reference)
3	Perioral numbness			
	61/70 (87.1)	60/89 (67.4)	23/45 (51.1)	4/70 (5.7)
	105 (30 - 368)	34 (11 - 105)	16 (4.7 - 52)	1 (reference)
4	Biting tongue			
	40/70 (57.1)	47/89 (52.8)	11/45 (24.4)	2/70 (2.9)
	47 (10 - 212)	40 (9 - 178)	12 (2.4 - 58)	1 (reference)
5	Hot sensation in the hand			
	47/70 (67.1)	50/89 (56.2)	17/45 (37.8)	1/70 (1.4)
	151 (19 - 1181)	97 (13 - 745)	45 (5.6 - 362)	1 (reference)
6	Hot sensation in the leg			
	44/70 (62.9)	38/89 (42.7)	13/45 (28.9)	1/70 (1.4)
	110 (14 - 853)	48 (6.4 - 369)	26 (3.3 - 215)	1 (reference)
7	No pain when burn or wounded**			
	42/70 (60)	44/89 (49.4)	6/45 (13.3)	0/70 (0)
	110 (14 - 852)	68 (8.9 - 522)	11.9 (1.4 - 104)	1 (reference)
8	Difficulty in judging the adequate temperature of bath water			
	38/70 (54.3)	31/89 (34.8)	6/45 (13.3)	1/70 (1.4)
	78 (10 - 601)	36 (4.7 - 273)	9.9 (1.1 - 86)	1 (reference)
9	Hanging a bag with elbow or shoulder instead of holding it in your hand			
	51/70 (72.9)	53/89 (59.6)	15/45 (33.3)	7/70 (10)

		30 (11 - 83)	16 (6.4 - 42)	5.6 (2.0 - 16)	1 (reference)
10	Headache				
		60/70 (85.7)	57/89 (64)	27/45 (60)	11/70 (15.7)
		53 (18 - 153)	16 (6.5 - 41)	13 (4.8 - 37)	1 (reference)
11	Heavy sensation of head				
		58/70 (82.9)	62/89 (69.7)	25/45 (55.6)	8/70 (11.4)
		40 (15 - 112)	20 (7.7 - 50)	11 (3.9 - 29)	1 (reference)
12	Shoulder stiffness				
		64/70 (91.4)	73/89 (82)	37/44 (84.1)	33/70 (47.1)
		15 (5.6 - 43)	7.0 (3.1 - 15)	7.7 (2.8 - 21)	1 (reference)
13	Lower back pain				
		64/70 (91.4)	77/89 (86.5)	39/45 (86.7)	38/70 (54.3)
		11 (4.0 - 31)	7.1 (3.1 - 17)	7.2 (2.5 - 21)	1 (reference)
14	Muscle cramps				
		62/70 (88.6)	80/89 (89.9)	35/45 (77.8)	25/70 (35.7)
		15 (5.8 - 37)	18 (7.3 - 45)	6.5 (2.6 - 16)	1 (reference)
15	Disturbed vision				
		61/70 (87.1)	69/89 (77.5)	29/45 (64.4)	18/70 (25.7)
		21 (8.5 - 54)	11 (4.9 - 24)	5.8 (2.4 - 14)	1 (reference)
16	Limited peripheral vision				
		58/70 (82.9)	58/89 (65.2)	20/45 (44.4)	5/70 (7.1)
		72 (22 - 239)	27 (8.9 - 83)	12 (3.6 - 39)	1 (reference)
17	Difficulty in recognizing a thing in your sight when you continue to stare it				
		54/70 (77.1)	56/89 (62.9)	14/45 (31.1)	4/70 (5.7)
		59 (18 - 195)	29 (9.3 - 89)	7.9 (2.3 - 27)	1 (reference)
18	Difficulty in finding a good in the shop				
		55/70 (78.6)	57/89 (64)	23/45 (51.1)	12/70 (17.1)
		21 (8.5 - 52)	9.8 (4.3 - 22)	6.2 (2.5 - 16)	1 (reference)
19	Surprised by a car coming out from the side				
		56/70 (80)	57/89 (64)	23/45 (51.1)	13/70 (18.6)
		20 (8.1 - 49)	8.7 (3.9 - 19)	5.1 (2.1 - 13)	1 (reference)
20	Eyes are fatigable				
		64/70 (91.4)	76/89 (85.4)	35/45 (77.8)	30/70 (42.9)
		15 (5.5 - 41)	8 (3.6 - 18)	5.2 (2.1 - 13)	1 (reference)
21	Difficulty in hearing				
		55/70 (78.6)	60/89 (67.4)	22/45 (48.9)	28/70 (40)
		6 (2.7 - 13)	3.2 (1.6 - 6.4)	1.5 (0.7 - 3.3)	1 (reference)
22	Difficulty in understanding a word or a sentence even if you can hear it				
		47/70 (67.1)	43/89 (48.3)	13/45 (28.9)	4/69 (5.8)
		37 (12 - 119)	16 (5 - 48)	7.3 (2.1 - 25)	1 (reference)
23	Tinnitus				
		57/70 (81.4)	59/89 (66.3)	22/45 (48.9)	11/70 (15.7)
		19 (7.9 - 47)	8.6 (3.9 - 19)	4.1 (1.7 - 10)	1 (reference)

24	Difficulty in smelling				
		49/70 (70)	47/89 (52.8)	16/45 (35.6)	6/70 (8.6)
		27 (9.3 - 78)	13 (4.7 - 36)	6.4 (2.1 - 20)	1 (reference)
25	Difficulty in tasting**				
		46/70 (65.7)	38/89 (42.7)	10/45 (22.2)	0/70 (0)
		130 (17 - 1008)	49 (6.5 - 375)	20 (2.4 - 166)	1 (reference)
26	Difficulty in judging the taste of your own cooking**				
		46/70 (65.7)	39/89 (43.8)	13/45 (28.9)	0/70 (0)
		130 (17 - 1004)	53 (6.9 - 400)	28 (3.5 - 230)	1 (reference)
27	Stumbling on flat ground				
		58/70 (82.9)	58/89 (65.2)	18/45 (40)	4/70 (5.7)
		150 (38 - 596)	52 (14.5 - 190)	21 (5.3 - 80)	1 (reference)
28	Stumbling tendency				
		64/70 (91.4)	71/89 (79.8)	27/45 (60)	10/70 (14.3)
		107 (32 - 353)	37 (14 - 99)	16 (5.5 - 44)	1 (reference)
29	Staggering				
		58/70 (82.9)	60/89 (67.4)	28/45 (62.2)	10/70 (14.3)
		34 (13 - 90)	14 (5.9 - 33)	12 (4.5 - 32)	1 (reference)
30	Difficulty in wearing slippers				
		53/70 (75.7)	52/89 (58.4)	13/45 (28.9)	2/70 (2.9)
		131 (28 - 618)	56 (12.4 - 251)	16 (3.3 - 79)	1 (reference)
31	Coming off your slippers or sandals while walking				
		53/70 (75.7)	46/89 (51.7)	15/45 (33.3)	3/70 (4.3)
		73 (20 - 271)	24 (6.8 - 85)	11 (2.8 - 42)	1 (reference)
32	Difficulty in fine finger task				
		56/70 (80)	64/89 (71.9)	25/45 (55.6)	4/70 (5.7)
		100 (26 - 383)	64 (17 - 232)	30 (7.8 - 116)	1 (reference)
33	Difficulty in buttoning				
		51/70 (72.9)	46/89 (51.7)	16/45 (35.6)	2/70 (2.9)
		101 (22 - 468)	38 (8.4 - 167)	20 (4.3 - 98)	1 (reference)
34	Dropping things held in the hand				
		53/70 (75.7)	60/89 (67.4)	18/45 (40)	7/70 (10)
		43 (14 - 128)	27 (9.5 - 77)	10 (3.1 - 29)	1 (reference)
35	Dropping chopsticks while eating				
		53/70 (75.7)	51/89 (57.3)	14/45 (31.1)	4/70 (5.7)
		50 (15 - 161)	21 (6.7 - 63)	7.2 (2.1 - 24)	1 (reference)
36	Difficulty in speaking words or sentences well				
		50/70 (71.4)	47/89 (52.8)	13/45 (28.9)	3/70 (4.3)
		54 (15 - 196)	24 (6.9 - 84)	8.9 (2.3 - 34)	1 (reference)
37	Hand weakness				
		55/70 (78.6)	67/89 (75.3)	20/45 (44.4)	9/70 (12.9)
		35 (13 - 95)	29 (11 - 76)	7.7 (2.8 - 21)	1 (reference)
38	Leg weakness				

		60/70 (85.7)	61/89 (68.5)	21/45 (46.7)	10/70 (14.3)
		63 (21 - 186)	21 (8.1 - 52)	9.2 (3.3 - 25)	1 (reference)
39	Hand tremor while moving				
		52/70 (74.3)	56/89 (62.9)	17/45 (37.8)	6/70 (8.6)
		34 (12 - 100)	19 (6.7 - 52)	7.2 (2.3 - 22)	1 (reference)
40	Postural hand tremor				
		46/70 (65.7)	44/89 (49.4)	9/45 (20)	4/70 (5.7)
		45 (13 - 165)	22 (6.3 - 78)	5.9 (1.5 - 24)	1 (reference)
41	Vertigo (feeling of spinning around)				
		47/70 (67.1)	50/89 (56.2)	18/45 (40)	4/70 (5.7)
		40 (11 - 144)	26 (7.4 - 89)	13 (3.5 - 49)	1 (reference)
42	Swaying dizziness				
		44/70 (62.9)	43/89 (48.3)	15/45 (33.3)	3/70 (4.3)
		60 (13 - 270)	33 (7.4 - 146)	17 (3.6 - 83)	1 (reference)
43	Fainting (syncope like) dizziness				
		34/70 (48.6)	28/89 (31.5)	11/45 (24.4)	3/70 (4.3)
		30 (7 - 136)	14 (3.2 - 62)	11 (2.2 - 52)	1 (reference)
44	Dizziness when standing up				
		60/70 (85.7)	74/89 (83.1)	27/45 (60)	10/70 (14.3)
		51 (17 - 151)	42 (15 - 116)	12 (4.1 - 34)	1 (reference)
45	General fatigue				
		63/70 (90)	68/89 (76.4)	36/45 (80)	24/70 (34.3)
		16 (6.1 - 41)	5.9 (2.8 - 12)	7.0 (2.8 - 18)	1 (reference)
46	Difficulty in sleeping				
		60/70 (85.7)	66/89 (74.2)	28/45 (62.2)	17/70 (24.3)
		22 (8.9 - 56)	11 (4.9 - 24)	6.4 (2.6 - 16)	1 (reference)
47	Appetite loss				
		47/70 (67.1)	37/89 (41.6)	17/45 (37.8)	3/70 (4.3)
		45 (12 - 161)	16 (4.4 - 54)	14 (3.6 - 52)	1 (reference)
48	Lack of motivation to do things				
		51/70 (72.9)	58/89 (65.2)	28/45 (62.2)	19/70 (27.1)
		7.6 (3.4 - 17)	5.3 (2.6 - 11)	5.0 (2.1 - 12)	1 (reference)
49	Cannot persevere or cannot keep working				
		52/70 (74.3)	53/89 (59.6)	20/45 (44.4)	10/70 (14.3)
		21.2 (8.4 - 54)	10.3 (4.4 - 24)	5.0 (2.3 - 16)	1 (reference)
50	Feeling as if your mind has become blank or empty				
		53/70 (75.7)	37/89 (41.6)	9/45 (20)	2/70 (2.9)
		130.9 (27.7 - 620)	26.4 (5.9 - 118)	5.0 (2 - 51)	1 (reference)
51	Cannot think about anything				
		47/70 (67.1)	35/89 (39.3)	10/45 (22.2)	1/70 (1.4)
		158.8 (20.2 - 1248)	46 (6 - 353)	5.0 (2.7 - 186)	1 (reference)
52	Losing your train of thought during conversation				
		56/70 (80)	55/89 (61.8)	20/45 (44.4)	10/70 (14.3)

		28 (11 - 71)	10 (4.5 - 24)	5.8 (2.2 - 15)	1 (reference)
53	Forgetfulness				
		67/70 (95.7)	78/89 (87.6)	36/45 (80)	48/70 (68.6)
		14 (3.8 - 53)	4.0 (1.7 - 10)	2.8 (1.0 - 7.8)	1 (reference)
54	Feeling as if you are not yourself				
		37/70 (52.9)	31/89 (34.8)	9/45 (20)	2/70 (2.9)
		39 (8.7 - 176)	4.0 (4.1 - 81)	9.2 (1.0 - 45.8)	1 (reference)
55	Irritation				
		59/70 (84.3)	62/88 (70.5)	30/45 (66.7)	17/70 (24.3)
		17 (7.1 - 41)	7.8 (3.6 - 17)	6.0 (2.5 - 14)	1 (reference)
56	Anxiety				
		57/70 (81.4)	57/89 (64)	27/45 (60)	18/70 (25.7)
		13 (5.8 - 32)	5.6 (2.7 - 12)	4.8 (2.0 - 11)	1 (reference)
57	Feeling sad				
		47/70 (67.1)	44/89 (49.4)	23/45 (51.1)	22/70 (31.4)
		4.5 (2.1 - 10)	2.1 (1.0 - 4.2)	2.4 (1.1 - 5.5)	1 (reference)
58	Difficulty in finding something when interrupted				
		55/70 (78.6)	61/89 (68.5)	22/45 (48.9)	18/70 (25.7)
		13 (5.4 - 29)	6.9 (3.2 - 15)	3.4 (1.4 - 8.0)	1 (reference)

* Adjusted for age, sex, either complication, and drinking history (including past); ** When prevalence of either district was zero, we postulated that a positive finding was found in the eldest subject in the district and calculated the OR and 95% confidence interval.
